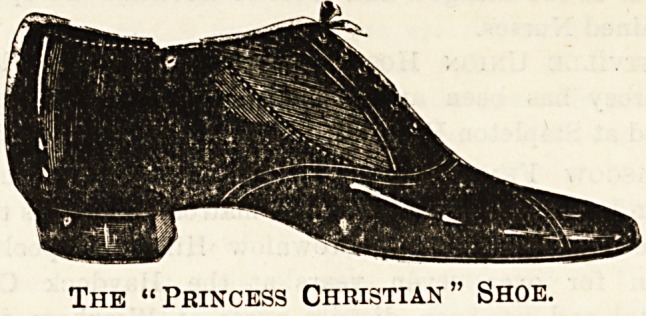# The Hospital. Nursing Section

**Published:** 1903-06-13

**Authors:** 


### The Hospital.

**Nursing Section.**

Contributions for this Section of "The Hospital" should be addressed to the Editor, "The Hospital" Nursing Section, 28 & 29 Southampton Street, Strand, London, W.C.

No. 872.—Vol. XXXIV. *SATURDAY, JUNE* 13, 1903.

### Notes on News from the Nursing World.

### THE PRINCESS OF WALES AND THE INFIRMARY PATIENTS.

Three years ago the Princess of Wales, then the Duchess of York, paid a quiet visit to the Portsmouth Workhouse Infirmary, so that she might see for herself how the guardians cared for their sick and infirm. Since then the Princess has not forgotten the little episode, and Miss Young—one of 30 infirm and bedridden females in ward 18—now in her 86th year, has been made happy each Christmas by receiving a card from her Royal Highness. Last month, after consultation with the doctor, Miss Young had her photograph taken and, with a letter of loyal good wishes, sent it to Marlborough House on the birthday of the Princess of Wales. Moreover, ward 18 had a birthday feast of its own to celebrate the event. The table was garnished with flowers, the guests included all whom the Royal visitor had especially noticed, the good health of the Princess was drunk in tea, and the nurses dispensed cake, which they somehow procured from somewhere. Next day came a gracious message from her Royal Highness with thanks for the photograph, followed by an announcement that the Princess was sending a piece of her birthday cake. It arrived on June 3rd the date of the Prince of Wales's birthday. There was then another birthday party, more cake was provided, and the Royal gift was divided between 36 tea drinkers, whilst the boys of the Children's Home played "God Bless the Prince of Wales" below the windows.

### NARROW ESCAPE OF A SISTER AT PRETORIA.

During the past few months nurses in South Africa have suffered considerably from the intensity of the storms, especially at the camps at Pretoria. It is nothing unusual for a sister coming back from duty to discover her tent, and all its contents, bed and belongings, etc., soaked through, or to get drenched in crossing from the hospital to her quarters, and to find nothing dry enough to put on when she gets there. A sister had a narrow escape a short time ago. She had barely stepped outside of her tent when the whole thing was lifted up on the wings of the storm, and dashed to pieces; half a second sooner and she would have shared its fate. As it was she was flung to the ground and did not recover from the shock for some time.

### NUNS AS NURSES.

A return ordered by the House of Commons has just been published, showing that 415 nuns are employed in workhouse infirmaries in Ireland. Of these, 32 are untrained, 48 schoolmistresses, and 335 nurses. The amount which they receive annually in salaries is £13,504. Fifty nuns are employed in the two Dublin unions, 13 in Cork infirmaries, nine in Limerick, 10 in Waterford, six in Trim, five in Baltinglass, six in Dungarvan, 10 in Enniscorthy, five in Galway, eight in Kilkenny, 10 in New Ross, eight in Killarney, six in Rathdrum, four in Navan, six in Youghall, six in Thomastown, and five in Glen. Assuming the accuracy of these figures, the plea of so many of the Irish Boards of Guardians that they cannot afford to pay fully-trained nurses does not seem tenable.

### A TRAINED NURSE FOR GRANARD INFIRMARY.

As we anticipated, Dr. Kenny has denied *in toto* all the charges of discourtesy and insult made against him by the nuns at the Granard Workhouse Infirmary, and intimated that he is most anxious to promote harmony combined with efficiency amongst the officers. The Irish Local Government Board inspector has also been acting as peacemaker, and at his suggestion the two nuns have consented to stay on as nurses until other arrangements have been made. The fact that the guardians have decided to appoint a fully-qualified nurse is, however, the most hopeful and practical sign that the nursing will be placed on a more satisfactory footing.

### HEROISM OF A BIRMINGHAM NURSE.

Last week a gallant attempt was made by Miss Lindsay, a Birmingham nurse, to save the life of a patient under her charge who was suffering from mental affliction. The patient was Miss Deykin, the daughter of a Birmingham manufacturer, who had been sent to Bromsgrove for change of air under the care of Miss Lindsay and another nurse. Miss Lindsay, missing her from home, went in search of her and found her sitting on a stile near the railway level crossing. She readily consented to return home, but on the way back suddenly ran off as fast as she could towards the railway. The nurse pursued her, and caught her at the metals just as the express train from Bristol was seen in the distance. A desperate struggle ensued, and at last by a violent wrench Miss Deykin got free from the nurse, threw herself in front of the train and was cut to pieces on the spot. The engine struck the nurse and she only narrowly escaped death. She was placed in the train at Bromsgrove and taken to the Queen's Hospital, Birmingham, of whose private nursing staff she is a member. We have since learnt that Miss Lindsay was trained at the Queen's Hospital, and is now in her fourth year. It is hoped that her injury is not serious, and we hear with much pleasure that though she is still of course suffering from severe shock, she is progressing as favourably as can be expected in the circumstances. Her heroic effort to save her patient has naturally excited the greatest interest and sympathy.

### A VISITING NURSE IN MINNESOTA.

Some interesting details are given of the result of the experiment of running a visiting nurse in the city of Minnesota, U.S. The record of her labours for the first three months is that she visited and cared for 28 patients, paying them 258 visits, and giving them such attention in their homes as the case required, or as the doctor prescribed. In many cases she made two and three visits a day, and in very severe cases she remained the whole of the night. The association under which she carries on her profession has a loan closet, which is furnished by the guilds of the various churches, and contains such articles as sheets, pillows, slips, towels, night-dresses, and infants' outfits. The nurse is allowed to use these at her discretion for the comfort of her patients during their illness, the only stipulation being that the articles are to be returned clean. The transformation in the homes of some of the very poor since the association and its nurse have been at work has already been so great that it is proposed to appoint a second nurse who will be allowed to visit cases of infectious diseases.

### MARRIED BY DECLARATION.

A strange case, bearing upon the peculiarities of the Scottish marriage law, was heard in the Court of Session, Edinburgh, last week, before Lord Stormonth-Darling. Miss Helen Steel Dykes, who in 1895 was a nurse at the Western Infirmary, Glasgow, brought an action against Mr. Joseph Muir Corbett, M.B., for "declarator of marriage." The plaintiff and the defendant had become engaged, and on May 21st, 1896, Miss Dykes received a letter from Mr. Corbett enclosing a declaration of marriage. In this letter he said that he wished Miss Dykes to have an absolute hold of him, and for that purpose enclosed the document. He added that he had consulted the necessary authorities on the subject, and made sure that the document would stand good. The declaration was in the defendant's handwriting, and bore his signature. The plaintiff appended her signature, and when she saw the defendant at the infirmary next day showed him the document with the two signatures, both understanding that they thus took each other as man and wife, though they agreed still to behave as engaged people only. It was further decided that they should be publicly married in church when Mr. Corbett could afford to keep his wife. The defendant now refused to go through the ceremony, though the Lord Advocate had decreed that he should do so within three months. Lord Stormonth-Darling granted the declarator, and ordered Mr. Corbett to "adhere to the plaintiff," or failing, to pay the agreed sum of £25 per annum.

### THE NEW SUPERINTENDENT OF DUDLEY INFIRMARY.

The post of superintendent nurse at Dudley Union Infirmary has been accepted by Miss Elizabeth Wilson, of Christchurch Union Infirmary. Prior to the appointment being made, a deputation of the Dudley guardians visited Christchurch Infirmary and pursued inquiries on the spot as to the qualifications of Miss Wilson. They reported that they were satisfied that she is in every way qualified for the position. We hope that Miss Wilson will therefore have their full support in the difficult duties which she will have to discharge at Dudley. Miss Newbury, her predecessor, in her efforts to improve the nursing, had to carry on a constant warfare with the guardians, and, in spite of the backing which she received from the Local Government Board, she was at last obliged to relinquish the contest. Miss Wilson may have the pleasure of reaping where Miss Newbury has sown.

### THE "LEICESTER NURSES' HOME."

It is expected that the new "Leicester Nurses' Home" of the Norfolk and Norwich Hospital will be opened by the Countess of Leicester on July 16th. The home contains about 70 bedrooms, for the whole of the nursing staff, including the private nurses. It has also two very large sitting-rooms, one of which will be used as a writing room and library.

### NURSING AND CRICKET.

Unlike some of our fashionable watering-places, Harrogate does not expose itself to the reproach of failing to supply the funds necessary for the nursing of the sick poor. The District Nursing Society has now been established seven years, and the report, which was adopted at the annual meeting the other day, shows that in 1902 the receipts exceeded the expenditure to the extent of £73. The number of cases nursed was 139, and the number of visits paid 3,037. The Mayor, in congratulating the society upon its success, judiciously urged that there should be no slackening on the part of the collectors, and appealed to the cricket club to get up a match to assist the endowment fund. This is an excellent idea, and now that the cricket season is in full swing all over the country, we hope that many local contests may be arranged in the interests of the nursing organisations. The votaries of football have done their share fairly well, and cricketers, we are sure, will not wish to be in any way behind them.

### A HOME OF REST FOR NURSES.

The committee of the Almondsbury Memorial Institute and Hospital, near Bristol, have made arrangements by which they can receive four or five nurses for a pleasant holiday or for rest and quiet during convalescence. The Memorial Institute is a fine building in a beautiful part of the country, erected by Mr. Sholto Vere Hare in memory of his wife 10 years ago. There are more rooms than can be occupied by the parochial charities, etc., for which the institute was originally intended, and Mrs. Chester-Master, the wife of Colonel Chester-Master, has therefore determined that nurses shall have the benefit of the spare apartments. Bristol nurses have the first claim, and are received for 10s. a week, or 5s. for the "week-end." Nurses from other parts pay 15s. to £1 per week. There are three very nice rooms for the nurse visitors, who are fetched from the station—Patchway, on the G.W.R.—by a waggonette for 1s. 6d. Knole Park, the lovely demesne which surrounds the residence of Mrs. Chester-Master, is always open to them, and cheap excursions can be taken up the Wye Valley to Tintern Abbey, Chepstow, Raglan and Berkeley Castles, whilst the roads for cycling are admirable.

### THE VALUE OF LADY COLLECTORS.

At the annual meeting of the Dunoon District Nursing Association, the chairman and other speakers bore testimony to the value of the services of the lady collectors, without whom, it was said, it would be almost impossible to obtain the money needed for carrying on the work of the organisation. The ideal state of things would, of course, be for people to send in sufficient money without any need of a reminder that it was wanted. But so long as such an ideal is not capable of realisation, collectors of energy and persistence are the back bone of a nursing association. The ladies of Dunoon pursue their labour of love through a wide district in spite of severe weather and occasional rebuffs, and thanks largely to their efforts, the poor are able to enjoy the benefits of being attended by a trained nurse.

### THE REORGANISATION OF BILSTON NURSING ASSOCIATION.

At a meeting in Bilston which was held for the purpose of considering the proposed reorganisation of the District Nursing Association, it was suggested that the spare time of nurses might be utilised for paying cases. Apart from the fact that as there are only two nurses who make an average number of ten visits per day, including Sundays, there can scarcely be any spare time to utilise, we advise the committee not to enter upon a policy which often tends to diminish rather than to augment the funds. To add paying cases would render the task of reorganisation more difficult because it could not then be pleaded with so much force that the Committee were asking for money to run a charity. But it is important that the old foundation, according to which 25 individuals are at present responsible for the money needed, should be altered, and that the people of the town generally should provide the sum requisite to carry on the Association. It is intimated that the annual cost of each nurse is £110, and Bilston will therefore be called upon before the end of the year, when the original arrangement lapses, to subscribe £220, half a guinea qualifying for membership and £2 2s. to a seat on the general committee. Bilston is sufficiently flourishing to warrant the expectation that now the situation has been faced, there will be no trouble about obtaining the cash.

### HOSPITAL LINEN.

A practical article upon hospital linen appears in the current number of an American periodical. The hints to those in charge of store-rooms are distinctly valuable, but there are also details which are of general application. Economy in the use of hospital linen is insisted upon as an important part of the nurse's training which will benefit her through life. It is pointed out that the nurses are in no small measure responsible for the appearance of the linen; that blood-stains or other stains can be readily washed out when they are fresh, and that every nurse should be taught that it is her duty to remove stains as far as possible by a preliminary soaking and washing before being sent to mingle with other linen in the laundry. If such matters were thoroughly impressed on each nurse as part of her hospital training there would, it is maintained, be fewer complaints of nurses in private practice needing an extra maid to wait on them. With regard to personal linen, each nurse, it is suggested, should bring with her two laundry bags, one to be kept in her room and receive soiled linen, the other to be sent to the sorting-room to remain till the clean clothes are returned in it. A list of articles sent should be attached to the bag. Everything should be marked in full, and a uniform system of marking should exist throughout the staff, so that the sorter should know at once where to look for the name and not have to turn each garment over a dozen times before finding what is required. As a means of curing carelessness, it is remarked that if linen not marked in full and in the right place were returned to the sender unwashed, there would soon be an improvement.

### SUNBEAMS AT CHATHAM.

At the annual meeting of the Chatham Queen Victoria Jubilee Nursing Association Mr. George Winch, in moving the adoption of the report, which showed that close upon ten thousand visits were paid by the nurses, told his hearers that "the ten thousand visits meant ten thousand sunbeams to light up the homes of the sick in Chatham." A new departure, on which he commented with approval, is an arrangement which has recently been entered into with the Royal Marines by which the association will receive £40 a year on the understanding that a nurse is always at the disposal of the wives and families of marines. It is expected that during next year a suitable nurses' home, for which a site has already been given, will be provided, and when this is done it is intended to obtain the services of a trained maternity nurse. The balance in hand at the end of the year was between £12 and £13, but if the work continues to increase in the same ratio as it has done during the four years of its existence, the staff will require to be augmented.

### A START AT STONE.

It was only at the end of last year that a nursing association was formed at Stone, and the Queen's nurse who was appointed did not begin her duties in the parish until the end of March. She has since attended 28 cases and paid upwards of 600 visits. Her work has already excited so much interest that a concert on behalf of the fund has just realised from £10 to £12, and it is intended to hold a house-to-house collection in June for the same object. The committee had previously raised upwards of £60 for this year's expenses, and it looks as if the nursing fund would be adequately maintained.

### SHORT ITEMS.

The Halifax Guardians have increased the salary of Miss Frost, matron at the workhouse infirmary, from £80 to £100 a year, and that of Miss Ward, the assistant matron, from £40 to £45 a year.—The s.s. *Arissa*, which arrived [illegible] [illegible]uthampton last week, had on board Nursing [illegible]r Bond, of the Army Nursing Service, who h[illegible] [illegible]ome home from South Africa on leave for te[illegible] [illegible]eeks.—Last week Miss Ellen Ellis, of the Blackheath Nursing Institution, received the silver medal which is presented to members of the staff for five years' faithful work. She is remaining on the staff.—Two of the nurses of St. Luke's Hospital, Halifax—Nurses Milner and Symons—have been up for their L.O.S. examination, and both were successful.

### The Nursing Outlook.

> " From magnanimity, all fear above;
> From nobler recompense, above applause,
> Which owes to man's short outlook all its charm."

### THE SERVANT OF THE POOR.

THE Princess of Wales this week visited one of the poorest and most deserving of our maternity hospitals in order to open a new nursing home. The hospital itself is small, and the staff of nurses might seem high, did one not take into consideration the large amount of outdoor work that is done. The public that subscribes knows but little of this outdoor work, and yet we believe that no nurses render greater service than the maternity and midwife pupils who go amongst the poor in their own homes—who attend not only at the labour, but visit afterwards and dress the baby and wash the mother, and do their best to give our wee citizens of the future a fair start in life. Just now the subject of the proper training for these nurses and midwives is much to the fore, and it would be well before any foolish plans are rushed upon, if the Hospitals Association would invite all the lecturers and matrons of the London maternity hospitals to a conference on the subject. Let it be granted at once, as we have frequently pointed out, that the training in the past has left much to be desired; but let it also be granted that Miss Gregory's proposal to build a new maternity hospital with a course of two years' training is fraught with many difficulties. However much we may regret that the existing hospitals have not been more strict in their course, we feel that the public owe them a debt and that it would be both wiser and kinder to set existing institutions in order, instead of starting new ones, to divert subscriptions and add to charity's burden. The maternity nurse and the midwife get trained in hospital to the use of endless disinfectants, clean linen, and long rules; and they are then sent out in the district and trained to do without all these things. In hospital some 50 articles have to be put ready for each labour; on the district we have known instances when there was but one article—and that a beer-can!—available for every purpose. This is excellent practice—to learn first how things should be done, and then how they can be done, and if only sufficient time were given we can see nothing w[illegible]ng with the method. Besides the poor greatly appre[illegible] the services of the nurses and midwives; if a [illegible]r attends he cannot be expected to wash the [illegible]by! Therefore we are heartily glad of the visit of the Princess of Wales to the pretty, clean little home for 20 nurses in Endell Street, and we wish the British Lying-in Hospital and its compeers all success.

Now if the midwife and the monthly nurse are still to remain the servants of the poor, are still to take their training in the London slums, there must be no building of some gorgeous hospital in an outlying district—no creating of a new order of nurses. It would be amply sufficient to lengthen the course of training in the present hospitals, to improve the nursing homes as the authorities in Endell Street have done, and to try and bring the public to understand and sympathise with the work of the nurse who looks after the very poorest mothers in the land. Surely Queen Charlotte's Hospital, after all these many years, deserves the title of " National Training School for Midwives ? " It has gradually been increasing its staff, and increasing the status of its staff, and the value of its certificate; there is still room for improvement; the old monthly nurse must go altogether shortly—must be replaced either by the fully-trained nurse or the midwife acting as nurse. But the term—six months—for midwifery at the proposed new establishment is the same as the proposed term at Queen Charlotte's and elsewhere. The only new point is the suggested first 18 months of " general and maternity " nursing. But the medical profession will never acknowledge a hospital to which general and maternity cases are both admitted; we have at least got a step beyond that in our knowledge of hygiene. Granted general training is a *sine quâ non*, it should be taken in a general hospital. We sympathise very warmly with Miss Gregory's desire to improve the midwifery teaching of the day, but we hope that at the meeting next week there will be free and open discussion and an attempt made to really find out which is the wisest way to obtain the object in view. To set up a personal hobby and ride for it rough-shod is apt to lead to ultimate failure, though it may secure attention for the time being. There are the long years to come to be considered, and the harassed treasurers of present hospitals might well give hints of the difficulties of money-gathering, of keeping up with the times and of keeping in the public eye. It always appeals more to the ambitious to start a scheme of their own than to work in with the existing state of affairs—to try and amend the things which are. But it is impossible to see any reason for a brand-new hospital founded solely for the training of a new form of midwife—founded apparently without any thought of the patients or the medical men. It makes no keen call on our sympathy. As a professional woman the midwife should make no demand on charity; she should pay for her training—as she does nowadays—and she should take her training where it suits the patients, and as it suits the medical men. When we consider the quiet, excellent work done both in the wards and in the district by such institutions as the General and the City of London Lying-in Hospitals, besides those we have already mentioned, when we think of the nurses at all hours of the day and night going into the slums of Shoreditch or Southwark, and in vermin-infested rooms, without even at times warm water to help them, doing their best for mother and babe, we regret the slight on them that we feel is made (even if it be not meant) by these rash new proposals, and we thank a thousand times the Princess who has shown her sympathy with the maternity hospitals of London at this trying time.

### Lectures on Ophthalmic Nursing.

By A. S. Cobbledick, M.D., B.S.Lond., Senior Clinical Assistant Royal Eye Hospital, late House-Surgeon and Registrar, Royal Eye Hospital.

### LECTURE XII.—ACUTE PURULENT CONJUNCTIVITIS—CAUSATION, SYMPTOMS, PROGNOSIS, AND TREATMENT.

This is a much more serious condition than the form described in the last lecture.

It is characterised by more intense initial symptoms, followed by a copious yellow discharge and swelling of the conjunctiva and eyelids. The risk of serious complications is present in all cases unless treatment is early and energetic.

*The Causation.*—This varies, but in all cases it is due to known organisms—cocci or bacilli—infecting the conjunctival sac.

1. It is a common affection in new-born babies, and is termed *ophthalmia neonatorum.* The intensity of this disease varies; the worst cases are due to the gonococcus, and infection takes place during the birth of the child. Less intense cases are due to infection from other organisms than the gonococcus, and infection also takes place during birth.

2. Ophthalmia due to the gonococcus may also affect adults.

3. Accidental introduction of pus organisms; the exact source is in many cases difficult to trace.

4. Diphtheritic conjunctivitis. This is due to infection with the Loeffler diphtheria bacillus; isolated cases may be caused by a patient suffering from faucial diphtheria coughing in the surgeon's face. It is very seldom seen in epidemic form. The infection is a mixed one, *i.e.* pus organisms—streptococci—are present with the Loeffler bacillus, and to these the purulent discharge, present in the second stage of the disease, is due.

5. A purulent conjunctivitis is frequently seen in children, associated with discharge from the ears and a pustular eruption on the face; it follows measles, and less frequently whooping cough and scarlet fever.

*Symptoms.*—Slight cases. These begin with itching and pricking sensations in the lids; for a day or two there is a serous discharge and slight swelling of the lids. At the end of four or five days from the time of infection a purulent yellow discharge makes its appearance; the palpebral conjunctiva is red and velvety in appearance, but the ocular conjunctiva is not affected; such an attack runs a course of from a week to ten days.

In more severe cases, the onset is more sudden and intense; there is a sensation of a foreign body in the eye, with lachrymation, and severe burning and pricking sensations; the lids soon become red and swollen, and on examining the palpebral conjunctiva it is found to be red, and there may be small dark streaks due to thrombosis of some of the blood-vessels. The swelling of the lids increases in 12 hours, and the skin becomes dusky red and shiny; a serous discharge gumming the lids together precedes, for two or three days, the purulent stage. The ocular conjunctiva next becomes affected, the blood-vessels are engorged, so that the white sclerotic becomes red, the conjunctiva swells and becomes œdematous (chemosis) to such an extent that it may overlap the cornea and strangle the vessels supplying it with nutrition; in this manner there is a risk of the cornea sloughing; this is most likely to occur in severe cases of ophthalmia neonatorum and in diphtheritic conjunctivitis.

When the purulent discharge is established, it is very copious and seems to form as quickly as it is removed; unless some means of preventing adherence of the lids is adopted, the pus is retained and much damage to the cornea may ensue. The lids, especially the upper, become more swollen, so that it becomes most difficult to obtain a view of the cornea, and there is a tendency for the upper to overlap the lower lid.

The great risk in this disease is involvement of the cornea:—

1. The most serious condition possible is sloughing of the cornea with subsequent disorganisation of the whole eye.

2. Perhaps the most common complication is a diffuse haziness of the cornea without any definite ulceration.

3. Ulcers of the cornea may form at its margin or towards the centre and have a tendency to perforate, with the result that the iris protrudes, and when the ulcer heals becomes more or less attached to the scar.

If there is much chemosis the marginal ulcers may be overlooked, on account of the swelling overlapping the cornea.

A severe case of purulent conjunctivitis, even without corneal complications runs about a four-weeks course.

*Diagnosis.*—This is never difficult, but it may not be easy to determine when and through what agency the infection took place.

In new-born babies it may be said that if pus makes its appearance in the sac from three to five days after birth,

Fig. 1.

the infection took place during birth. If the discharge appears after the fifth day it is probable that infection has been due to cleansing the face with soiled sponge or towel.

Diphtheritic conjunctivitis is not frequently met with; in the early stage, *i.e*, during the first week after infection, there is but little discharge, and the lids are very tense and hard and the conjunctiva pale rather than red; these points, with the presence of a possible source of infection should make the diagnosis fairly certain.

*Prognosis.*—In mild cases this is always good as there is no fear of corneal complication. In severe cases there must always be some anxiety, especially if the case has not come under observation early.

In the worst cases of ophthalmia neonatorum the infection is sometimes so intense that treatment is powerless to stay the mischief, with the result that both cornea may become opaque or the eyes disorganised, complete blindness being the result.

In diphtheritic conjunctivitis there is also a great risk during the first week of the cornea sloughing.

Still the majority of cases if seen in time and treated energetically clear up with no serious implication of the cornea.

*Treatment.*—As prevention is better than cure, much can be done by making a routine practice of washing the eyes of all new-born babies with an antiseptic lotion *e.g.*, 1 in 5,000 hydrarg. perchlor., and for two days instilling drops of silver nitrate solution (gr. i. or ii. or ℥i.).

In the early stage of an attack cold should be frequently applied either by iced boracic flaps or Lieter's tube (Fig. 1). Directly any discharge is noticed the conjunctival sac should be washed out frequently—every two hours—with boracic acid lotion (4 per cent.). If the discharge increases the washing should be repeated every hour, day and night, and the lids smeared with vaseline or boracic acid ointment to prevent the lids adhering and so causing rentention of the pus in the sac; at this stage silver salts, *e.g.* the nitrate (gr. x. or xv. or ℥i.), are invaluable applied once in 24 hours; the upper and lower lids should be everted and painted with the solution of silver nitrate; any excess of the solution must be neutralised with common salt solution.

If a child comes under observation at this stage, there are two important points for nurses and students to remember: 1. The lids may be firmly gummed together, and the sac be full of pus under considerable pressure, so that when the lids are opened the pus squirts out in a jet. Nurses who are continually attending to these cases in out-patient practice should therefore make a rule of wearing large circular spectacles to protect the eyes from the possibility of infection. 2. The lids may be so swollen that a view of the cornea is difficult. Where this is the case, persistent attempts to retract the lids with the fingers should not be made, for a deep ulcer of the cornea, near the point of perforating, may be present, and any undue pressure on the ball of the eye would produce perforation, and possibly the appearance of the lens and vitreous. It is therefore better, if one careful attempt to see the cornea is unsuccessful, to use a metal retractor, whereby all pressure used is exerted on the lid and not on the eye-ball.

### Royal British Nurses' Association.

### ANNUAL MEETING.

Princess Christian presided over the annual meeting of the Royal British Nurses' Association, which was held at the Imperial Institute on Saturday afternoon. Her Royal Highness was received by Mrs. Coster, Miss Thorold, Sir James Crichton Browne, Sir Dyce Duckworth, Mr. John Langton, hon. treasurer, and Dr. Comyns Berkeley, medical hon. secretary of the Association.

Mr. Langton, in proposing the adoption of the financial report, deplored the state of the finances of the Association, which owed £253 16s. 9d. to sundry creditors, and this was causing him grave anxiety. The nurses would have to learn to help themselves, since it could not be expected that the Association would always have a treasurer who would get them out of their financial difficulties. He rejoiced that the assets for benevolent purposes were represented by a sum of £3,928 7s. 1d., all of which had been raised since he had had the honour of being treasurer, and he took the opportunity of expressing his deep obligations to those who had worked so energetically for the best welfare of the Association. The sum of £70 18s. 9d. was distributed during the year in benevolent grants from the Helena Benevolent Fund; and the Settlement Fund, which was attracting paramount interest among the members, was now credited with £2,491 14s. 2d. Many houses had been inspected and building sites visited, but up to the present time with no definite success, although no opportunity of acquiring suitable sites or buildings had been overlooked. Either the houses had failed to come up to the standard required, or the price of the land had been beyond their means to purchase.

Dr. Calvert seconded and Sir James Crichton Browne supported the motion, which was carried.

Dr. Comyns Berkeley read the report for 1902, showing that 118 nurses had applied for registration, of whom 93 had been accepted; 105 new members had been elected, and 17 had withdrawn. There had been 7 deaths. The number of lady consuls at home and abroad was now 51; and there was increasing evidence of the value of their services in promoting a knowledge of the Association and in stimulating public opinion in favour of the efficient training of nurses. The Society of Chartered Nurses was increasing in prosperity and popularity, and the Auxiliary Society continued to make good progress, its position becoming more assured every month. Miss Newcombe having seconded, the report was adopted.

The subject of State registration for nurses was introduced by Miss James, who said that it would bring order out of chaos, and Miss Catherine J. Wood followed, who observed that nothing satisfactory could be done until the profession had made up its mind what was to be done and how it was to be done. On a show of hands it was decided that the subject was one that should be discussed by the Association.

Princess Christian expressed the opinion that the large hospitals, institutions, and nursing schools should come together and decide what they thought it was right the trained nurse ought to be. They could not ask the State to register nurses until there was agreement between the hospitals. There were many good nurses who had not had a long training, and they must be fair to them. That was what she was anxious about.

On the motion of the President, Mr. John Langton and Mr. Edward Fardon were unanimously elected Vice-Presidents of the Association, and the meeting closed with a vote of thanks to Princess Christian for her continued and untiring support and frequent attendance at meetings, proposed by Mr. Langton and seconded by Sir James Crichton Browne.

Many of the members subsequently visited the Earl's Court Exhibition, where a pleasant evening was spent.

### To Nurses.

We invite contributions from any of our readers, and shall be glad to pay for "Notes on News from the Nursing World," or for articles describing nursing experiences, or dealing with any nursing question from an original point of view. The minimum payment for contributions is 5s., but we welcome interesting contributions of a column, or a page, in length. It may be added that notices of appointments, entertainments, presentations, and deaths are not paid for, but that we are always glad to receive them. All rejected manuscripts are returned in due course, and all payments for manuscripts used are made as early as possible after the beginning of each quarter.

### The Princess of Wales and the Nurses of the British Lying-in Hospital.

### OPENING OF THE NEW HOME.

A ROYAL welcome was accorded to the Princess of Wales on Monday afternoon, on the occasion of her visit to the new Nurses' Home in Endell Street. St. Giles' was gay with flags, and festoons of roses were hung across the road between the home and the tall houses on the opposite side, and crowds of spectators lined the roadways. Punctually at half-past three the carriage containing the Princess, who was accompanied by the Countess of Airlie and the Hon. Alec Hood, drove up, and the Royal visitor was received in the entrance hall by Lord Kinnaird, Vice-President of the hospital, Mr. C. E. Turner, Chairman, and Mr. A. H. P. Strickland, Vice-Chairman, of the Board of Management, Sir John Williams, and the matron, Miss Gertrude Knott. After the preliminary presentations, a lovely bouquet of white lilies, lilies of the valley, and asparagus and maidenhair fern was offered by one of the daughters of the chairman, and graciously accepted by the Princess. Her Royal Highness, who wore a light lilac silk dress with deep lace insertions, and a white toque, was then invited to go over the new building, which she did under the guidance of the Chairman and Sir John Williams, preceded by the matron.

The tour of inspection, during which her Royal Highness expressed her admiration of the home and its arrangements, ended with the new dining-room, where the nurses, drawn up in line, made obeisance as the Princess entered. Here the invited guests were assembled, and her Royal Highness, having inquired how the tables were arranged for meals, said in clear tones, "I have great pleasure in declaring this home open."

A short dedicatory service was then read by Canon Covington, rector of the parish and rural dean, and all joined in repeating the Lord's Prayer.

A number of little children next presented pretty green satin purses, each containing not less than £5, to be devoted to paying off the debt on the building, and the Princess appeared greatly to enjoy this part of the ceremony, especially when a tiny baby in its nurse's arms, who might have been expected to cling to the dainty bag, gave it up after an anxious moment. One purse, presented by a little girl in white, was on behalf of the tradesmen connected with the hospital.

The two senior physicians having been presented, her Royal Highness, again preceded by Miss Knott, was conducted upstairs, round the dome of the dining-room, which was draped with the Union Jack and decorated with large palms and shrubs, into the wards, the first to be visited being the new ward on the ground floor, which has been made available by the removal of the nursing staff into the new building.

The members of the Board of Management and the Ladies' Committee, the physicians, the nurses, and the children followed her Royal Highness, and assembled in the Endell Street entrance in preparation for the departure of the Princess, which took place amid the cheers of the delighted spectators lining the street.

The visitors were subsequently shown over the new Home, which is now quite completed and in full occupation by the nurses and pupils.

### The Duchess of Albany Opens the Kingston Nursing Home.

ON Monday afternoon the Duchess of Albany, accompanied by her daughter, Princess Alice, visited Kingston-on-Thames in order to open the new nurses' home belonging to the Nursing Association. The adjoining house, which has been bought by the Association, provides additional accommodation for three or four nurses, and also for paying patients. There are two very pleasant sitting-rooms on the ground floor, one of which opens out of the little garden.

A large marquee was erected in the garden of the original premises, and there the visitors, who assembled in large numbers, awaited the arrival of the Royal party. This was announced by the playing of the National Anthem by the band of the East Surrey Regiment, who were stationed in the roadway. Her Royal Highness was received by the Mayor of Kingston, the vicar of Kingston, the matron of the institution (Miss Sawyer), and members of the committee. A short visit of inspection having been paid a service of dedication was conducted by the Vicar of Kingston. The Royal party then repaired to the garden, where they took their seats on the daïs at the upper end of the marquee. Two beautiful bouquets were presented to the Duchess and Princess Alice by the daughter of the Mayor and Miss Marjorie Kane. The next and most picturesque feature in the proceedings was the presentation to the Duchess of Albany of about forty purses, each containing not less than two guineas, by the children of the neighbourhood and some of the nurses of the Association. Among the children, who were all dressed in white, were some so tiny that the Duchess had to stoop very low in order to take the little red and gold purses so firmly clasped in their small hands.

The Mayor of Kingston then welcomed the Duchess of Albany back after her absence of three years, and thanked her on behalf of the Association for her visit to the Home.

Mr. Walter East, the president, recalled the previous visit paid by the Duchess nine years ago, and said that since that date the work of the Association had gone on increasing. The same could not, however, be said of the subscriptions, which showed no advance whatever. He hoped that the inhabitants of Kingston would realise their responsibilities in this respect, and that there would be a large increase in the number of subscriptions; and he went on to illustrate the work of the Association by means of a few figures. In 1890, the first year of their work, the nurses paid 4,462 visits. Last year the total reached was 15,868, besides 395 special visits, which made an average of 45 visits per day, Sundays and week-days.

The Duchess of Albany then declared the Home open and a little later the Royal party drove away.

### Where to Go.

GRAND BAZAAR IN AID OF THE ROYAL FREE HOSPITAL, PRINCE'S SKATING CLUB, KNIGHTSBRIDGE. — Thursday, June 11th, Friday, June 12th, at 2. Admission after 7 on Thursday, and after 5, on Friday 1s.

### Death in Our Ranks.

WE learn with regret of the death from typhoid fever of Sister Ada Mary Caunt. She passed away at her father's residence on Whit Sunday, having contracted the disease whilst in the execution of her duties at the Basford Sanatorium, Nottingham. Sister Caunt received her training at Wisbech.

### Everybody's Opinion.

### NUNS AS NURSES.

"A Trained Nurse" writes: With reference to your article on "Nuns as Nurses" I wish to call your attention to the fact that you are entirely wrong as to the order of nuns you criticise. Your paper has a large circulation, and in justice to the sisters of St. Vincent de Paul your article should be contradicted as far as they are concerned. They live and have ever lived up to the rules of their founder, whom you quote. There has never been a fault found with their nursing, as there is nothing which can be done for the sick which they will not do, and nowadays they number among their order several trained nurses. The nuns in the Granard Union are quite a different order and have nothing whatever to do with the Sisters of St. Vincent de Paul.

"Caleb J. Powell, F.R.C.S.," Surgeon, North Dublin Union Hospital, writes: In your edition of the 6th inst. you comment on the "surprise" of the Irish Local Government Board at discovering "the list of nursing duties that the nuns of the Granard Union Hospital gave as being outside their province as nurses in charge of sick wards," and incidentally you state that the Sisters of St. Vincent de Paul (Sisters of Charity) are in charge of this hospital. I am aware that it takes a great deal to "surprise" an editor, but will you be surprised when I inform you that the Sisters of Charity are not and never have been in charge of the hospital to which you refer? I have personal experience of the Sisters of Charity as nurses. With the exception of two lay nurses they constitute the entire nursing staff of the North Dublin Union Catholic Hospital, and I can inform you that they have no "list of nursing duties as being outside their province as nurses in charge of sick wards."

[We did not state that the Sisters of St. Vincent de Paul are in charge of Granard Union Infirmary, nor was the article in question directed at the nursing qualities of the Sisters of St. Vincent de Paul.—Ed. The Hospital.]

### THE USE OF THE SCALPEL IN AN EMERGENCY.

"Inquirer" writes: Regarding the nursing of tracheotomy cases, will you kindly inform me if it is usual for a nurse, without any authority or instructions from the doctor, to make use of a scalpel in an emergency. If so, in event of the patient's death, could she be charged with manslaughter? This question arises from an incident which occurred in a hospital of over 100 beds, where there are resident three qualified house surgeons. The tube was removed from a tracheotomy patient apparently convalescent. After 24 hours breathing became difficult. Twice during the night the house surgeon was called, but gave no orders excepting with regard to nourishment. Forty-five minutes after his second visit the patient appeared to suffocate. Artificial respiration was applied, the scab removed (with dilators) from the wound, and the house surgeon hastily summoned. On his arrival he severely censured the "night sister" for not having made use of the "knife," and after himself making the incision and inserting the tube, and seeing the patient well restored, he repeated that the knife should have been used pending his appearance!

[Certainly it would have been most inadvisable for a nurse to make use of a scalpel under the circumstances mentioned. —Ed. The Hospital.]

### SCHOOL NURSES AND EDUCATION.

"Educationist" writes: One reason why the school nurses should be supported by the State does not seem to have been mentioned by Sir Henry Burdett, yet it is of great importance. It is generally admitted that many of the poorer middle class, who now send their children to cheap private schools, would get better education for them at some of our State-aided elementary schools, where the teachers are thoroughly trained, and the educational appliances are sufficient. That in some cases children are sent elsewhere than to these schools—to small and inconvenient private houses, where the teachers have no qualification for their work—may be due, in some cases, to their parents' notions of gentility. But very often decent parents object, and rightly object, to sending their children to a school, however good it may be, where they must sit side by side with dirty school-fellows. They are too self-respecting to be lured by the fact that the education is free, and probably are not themselves sufficiently educated to know whether it is better or worse, but they know if their children come home with dirty heads, or contract ringworm or some similar disease. The result is that the very class who would profit most by receiving good free education content themselves often with a very inferior kind—the best they can afford, but not good at that—while large well-planned buildings, admirable appliances, and thoroughly-trained teachers are given to those who regard all education only as a sort of penal servitude, in which they do as little as they can, and from which they escape as soon as possible. If by raising the standard of cleanliness among the children who now go to our free elementary schools, they can draw into these a higher class of pupils, the School Nurses' Society will perform a national service, and may well be supported by the State.

### Novelties for Nurses.

By Our Shopping Correspondent.

### BOOTS AND SHOES FOR NURSES.

Messrs. Gooch's Stores, of 63 to 77 Brompton Road, S.W., whose attention has for many years been devoted to evolving hygienic shoes for use in hospital wards, have recently enlarged their premises, and I should advise any nurse who is about to purchase a new pair to go and see the "Princess Christian" and "Samaritan Ward" shoes made by this firm before making her choice. Flat foot is a complaint so common among those whose occupation entails many hours of standing, that it is well to guard against it by wearing a suitable shoe, and those made by Messrs. Gooch are specifically designed to prevent, as well as to minimise, the evil.

The "Princess Christian" Shoe.

Between the sole and the upper covering there is a strong spring, and a carefully-moulded sock which is so arranged as to give support just where it is needed, on the inner side of the instep. Of course one can buy a shaped sock and put it into an ordinary shoe, but it is obviously better to have the two combined, and the materials used in these shoes are of so good a quality that the slight additional cost will be true economy in the long run. Moreover, 12s. 9d., and this is the cost of the Princess Christian shoe, is after all a very moderate price to give. These shoes are made for putting on easily and quickly; they have an elastic side, but a permanently laced front, and have the appearance of an ordinary laced-up shoe. The Samaritan Ward shoes have double straps, and are also suitable for summer walking shoes. Both varieties have a rubber tip on the heel to render them noiseless. The shaped sock and spring can be fitted to boots as well as shoes, and if a visit is not possible, Messrs. Gooch's manager assures me that he will be pleased to carry out orders by post, provided that a specimen boot or shoe is sent for the size. Shoes of specially dressed leather are made for Colonial wear.

There is also a great variety of evening and dancing shoes just now on view, many of which are fascinatingly pretty; indeed, I think I was shown every conceivable kind that the most fastidious wearer could desire, with the single exception of the glass slipper of Cinderella.

### Appointments.

No charge is made for announcements under this head, and we are always glad to receive, and publish, appointments. But it is essential that in all cases the school of training should be given.]

Ancoats Hospital, Manchester.—Miss L. Taylor has been appointed sister of the women's wards, Miss Wilkinson sister of the children's ward, and Miss Wright theatre sister. They were all trained at Ancoats Hospital.

Aston Union Infirmary, Birmingham.—Miss Jessie Price has been appointed sister of the male wards. She was trained at the Salford Union Infirmary, Manchester.

Bedwelty Workhouse Infirmary, Tredegar.—Miss H. M. Butler has been appointed superintendent nurse. She was trained at the Sunderland Workhouse Infirmary, and was afterwards charge nurse at Town's Hospital, Glasgow. She has since been alternately charge nurse and night superintendent at the Rochdale Workhouse Infirmary.

Bradford Royal Infirmary.—Miss Elizabeth Hodges has been appointed lady superintendent. She was trained at Guy's Hospital, London, where she was afterwards night sister, sister of the operation department, and assistant matron. She has since been matron of Cumberland Infirmary, Carlisle.

Burnley Union Infirmary.—Miss Florence Midgley and Miss Jane Wood have been appointed charge nurses. They were both trained at Hull Workhouse Infirmary and have since been charge nurses at Prestwich Union Workhouse.

Dunoon District Cottage Hospital.—Miss J. Shaw Paterson has been appointed matron. She was trained at the Victoria Infirmary, Glasgow, where she was afterwards charge nurse. For the past two years she has been a member of the Glasgow and West of Scotland Co-operation of Trained Nurses.

Eastville Union Hospital, Bristol.—Miss Florence L. Purcey has been appointed assistant nurse. She was trained at Stapleton Hospital, and holds the L.O.S. certificate.

Glasgow Female Lock Hospital.—Miss Laura H. Wilson has been appointed nurse-matron. She was trained at the Parish Infirmary, Brownlow Hill, Liverpool; was matron for over seven years at the Haydock Cottage Hospital, and has been district nurse at Wrexham for the last eight and a-half years.

Hospital for City Police, Bishopsgate,—Miss Kate Farrance has been appointed matron, and Miss Ada J. Brown has been appointed nurse. Miss Farrance was trained at the London Hospital, Whitechapel Road, London, and has since been on the private staff of that institution for four years. Miss Brown was trained at the Central London Sick Asylum, and has since been staff nurse at Poplar Hospital.

Morecambe Sanatorium.—Mrs. Minnie Sutton has been appointed nurse-matron. She was trained at East Lancashire Infirmary, Blackburn, and was afterwards for two years at the North Staffordshire Institute, sister at Monsall Hospital, Manchester, and housekeeper at the City Hospital, Park Hill, Liverpool.

Porth Cottage Hospital.—Miss Blanche A. Bradbury has been appointed matron. She was trained at Burton-on-Trent General Infirmary, and has since been charge nurse at Clevedon Cottage Hospital, and nurse at Exton Hospital, Yorkshire.

Portsmouth Workhouse Infirmary.—Miss Mabel K. Herbert, Miss Gertrude E. Scott, and Miss Hannah F. Young have been appointed charge nurses. Miss Herbert was trained at St. Leonard's Infirmary, Shoreditch, where she has since been staff nurse. Miss Scott was trained at Durham County Hospital, and has since been staff nurse at the Homœopathic Hospital, Plymouth. Miss Young was trained at Birmingham Infirmary, and has since been charge nurse at Rotherham Infirmary.

Prudhoe Memorial Convalescent Home, Whitley Bay.—Miss Henrietta Preston has been appointed assistant matron. She was trained at the Hospital for Women, Liverpool, and the General Hospital, Stockport. She has since been charge nurse at Stroud General Hospital, and sister at the Convalescent Home, Parkwood, Swanley, Kent, for six years, and night sister for the past 15 months at St. Mark's Hospital, City Road, London.

Rondesbosch and Mowbray Cottage Hospital, Rosebank, Capetown.—Miss M. L. Muriel has been appointed matron. She was trained at St. Bartholomew's Hospital, London, and has since been sister at Gwelo Hospital, Rhodesia, on the staff of the Private Hospital, Bulawayo, for private nursing, and night sister in the same hospital for three years and a half. She holds the L.O.S. and Clapham School of Midwifery certificates.

Sculcoates Union Infirmary.—Mrs. Kathleen Blake has been appointed superintendent nurse. She was trained at St. George's Hospital, London, and has since been matron of the Oxygen Home, London, sister at Birmingham Infirmary, and head charge nurse at Sculcoates Union Infirmary.

Southwark Infirmary, East Dulwich.—Miss Minnie Thompson has been appointed sister. She was trained at Southwark Infirmary.

Stapleton Union Hospital, Bristol.—Miss Bertha F Bishop and Miss Amelia J. Tudball have been appointed assistant nurses. They were both trained at Stapleton Hospital and both hold the L.O.S. certificate.

---

Colne Jubilee Cottage Hospital.—Miss McRae, the new matron, writes to say that she was not district nurse at Newbury, but had on two occasions acted as matron's *locum tenens* at the District Hospital.

### TRAVEL NOTES AND QUERIES.

Inexpensive Quarters Abroad (B. E. M.).—I will send you the addresses you ask for by post, but must ask you not to pass them on to others, as they are meant only for those whose means are genuinely small and not for ordinary tourists.

Rouen, Caen, Caudebec, etc. (Margaret).—St. Servan you will find on the north coast of the department of Ille et Vilaine. Caudebec is on the Seine in Normandy, between Havre and Rouen. I think either of these places suitable, or Bruges in Belgium. Rouen is near to Caudebec, and you could go there several times if you like. I will give you the Convent address privately if you decide to go there, but it is very quiet and in the heart of the country, and has no facilities for getting about. I do not consider it so suitable for two young women. It does not trouble me at all, and I like to help tired nurses.

The Way to Venice (Piazza San Marco).—Cheapest route *viâ* Newhaven and Dieppe, Bale, Milan, and Verona; fare, second return, £8 14s. 10d. Staying at Lucerne two nights—one is not enough for comfort, and you see nothing—go to Hôtel de la Poste, ask for room on third floor. It should not be more than 2 francs, or 2.50. At Venice go to Hôtel Pension Kirsch Riva degli Schiavoni, ask for rooms on fourth floor, or, if full, try Pension Bril-Da-Rue Traghetto S. Gregorio, or Pension Lewald Fondamenta S. Vio, 743. All these will ask terms from 5 to 8 francs per day. Get your tickets from Cook or Gaze, and say you wish to break the journey at Lucerne and Paris. You can take 56 lbs. of luggage each, free. At Paris stop at Hôtel Britannique, Avenue Victoria. There is time before you start, so if you want further information write to me again.

### Echoes from the Outside World.

### Royal Visit to Ireland.

IT is officially announced that the King and Queen will visit Ireland in July. Their Majesties are to leave London on Monday, the 20th of next month, and will arrive at Kingstown on the following day. During their stay in the sister island the King and Queen will be guests of the Duke and Duchess of Devonshire, at Lismore Castle, County Waterford, one of the finest residences in Ireland. It dates back to the time of King John, and after being rebuilt and enlarged, withstood a siege in the Cromwellian epoch. The Duke of Devonshire's family came into possession of the castle in the middle of the eighteenth century, when the Duke of that day, who had been Lord Lieutenant of Ireland, received it as a gift. Since it has belonged to the present owner, great improvements have been made. The surrounding scenery is very fine.

### Officers' Widows and Daughters.

THE Queen has contributed the sum of £5,000 out of her war fund towards the extension of the scheme for the benefit of officers' widows and unmarried daughters whose incomes do not exceed £100 per annum, by the provision of apartments, rent free, on the same system as those at Hampton Court and Kensington Palaces are granted by the King. In 1899, when the project was initiated, sufficient funds were privately subscribed to provide for the temporary accommodation of the widows and daughters of twelve officers. In consequence of the high rent demanded for suitable apartments, and the uncertainty of their tenure, it has been decided at once to make a commencement by building, as funds are forthcoming, suites of self-contained apartments on a very desirable site which has been obtained at Wimbledon. How greatly the benefit of a free home, with no anxieties as to rent and taxes, to those who have a barely sufficient income for maintenance, is shown by the grateful letters received from the twelve ladies who have been provided for during the last three years. The Queen's approval of the extension, and her substantial contribution towards it, cannot fail to give the movement the impetus it both needs and merits.

### Terrible Collision between Steamers.

ON Sunday afternoon a disastrous collision between two steamers took place off the Iles Maire, involving the loss of more than one hundred lives. One of the vessels was the *Insulaire*, and the other, which sank, the *Liban*. The latter was built in England in 1883 and was a vessel of 3,000 tons. Only one boat was able to be lowered. As soon as news of the collision, which was witnessed by a pilot vessel, reached Marseilles the Mayor directed that arrangements suitable to the circumstances should be made. The pilot vessel saved 40 persons and picked up eight bodies, and another vessel saved 57 and picked up 21 bodies. The bodies on their arrival at Marseilles were removed to the morgue, and laid in the chapel of the Municipal Hospital. The boilers exploded as the *Liban* went down, and the most heartrending cries were heard, followed by silence. It is stated that not more than twenty minutes elapsed between the collision and the complete disappearance of the steamer, and a terrible feature of the catastrophe was that the awning which covered the deck before it occurred afterwards became a cage, dragging the passengers down into the sea as the vessel sank.

### His Majesty's Theatre.

ONE of the most interesting theatrical events of the season took place on Monday evening, when a special performance in aid of Guy's Hospital was given at His Majesty's Theatre. The King and Queen, the Prince and Princess of Wales, and their suites, the Prime Minister, and many other distinguished persons were present, the house being crowded. The pit was filled with nurses wearing the well-known white caps of Guy's. First on the programme was the poetical drama of "Flodden Field," by the Poet Laureate, who has not before appeared among acted dramatists. The play, which is in blank verse, is based on an episode in connection with the Scotch war in the early part of Henry VIII.'s reign, the principal personages being James IV., represented by Mr. Fred Terry; Lady Heron, who loves the Earl of Surrey, commander of the English forces, and flirts with James IV., by Miss Constance Collier; and Lord Surrey by Mr. Oscar Asche. Then came an adaptation of Mr. Rudyard Kipling's "The Man Who Was," in which Mr. Tree, as the broken-down soldier who returns to his regiment after 20 years' exile in Siberia, had a part that fitted him like a glove. The burden of the acting in this admirable piece fell almost entirely upon the actor-manager, but Mr. Edward Maurice was also excellent as Colonel Dirkovitch. At the end of the performance Mr. Tree announced that the handsome sum of £2,100 had been realised.

### The New Census Summary.

THE newly-issued summary tables of the census of 1901 contain some interesting details with regard to women. There are over a million more women than men in England at present, ignoring young children, and only reckoning those who are old enough to earn their own living. For every 29 unmarried women over ten years of age who do no work outside their own homes, 32 earn their own living. Among married women only one of seven earns an income for herself apart from her husband. As to the employments they follow, 7,000 women are chemists, nearly 4,000 butchers, 300 undertakers, and 12 shepherds. The Government employ 26,000 women, there are 352 women doctors or dentists, and 64,000 "care for the sick." Of those now in the workhouse 15,000 were originally domestic servants, 13,000 charwomen, and 624 sick nurses. With regard to matrimony the statistics show that very young husbands usually prefer wives older than themselves. One husband of 15 has married a girl of 17, and one lad of 17 a woman of 35. There are 30 girls of only 15 and 162 of 16 years who have already become wives. Roughly speaking, to every six married people throughout the country there are eleven unmarried and one widowed. There are also two widows for every widower.

### Pictures at the French Gallery.

ALTHOUGH natives of Berlin think very highly of Professor Adolf von Menzel, who is known as the "Little Excellency" —the adjective being especially applicable to one who is physically amongst the smallest of men—the works of the artist are almost unknown in this country, although he is an Honorary Foreign Member of our own Royal Academy. The Menzel Exhibition now being held at the French Gallery, Pall Mall, is therefore particularly interesting, being representative of 70 years of hard work. As long ago as 1838 he painted a picture of a mother and child; as recently as a month ago, in his eighty-eighth year, he added two pencil drawings to his collection, which show that the strength and vigour of the artist have in no way deteriorated. In pencil, chalk, and water-colour work, Menzel is equally at home, and though many of his productions are stored in the Berlin National Gallery, the greatest part remain in the artist's own portfolios. From these he has consented to lend a small collection for exhibition in this country, and nurses who love to enrich their knowledge of art should not miss the exhibition.

### For Reading to the Sick.

### THE WAYSIDE CROSS.

Silent we rested where a towering Cross
 On the dry fields of far Bavaria stands;
And wide as man's illimitable loss,
 Its all-embracing arms, like Love, expands.

O great Example!—superhuman tie
 Fashioned in Heaven! love-chant of many parts
By Angel chorus sung, while glad reply
 Echoes on earth from thousand bleeding hearts!

There still it stands no friendly form is nigh
 Only the wayworn pilgrim kneeling down
With head reclined, but tearful upward eye
 Forgetting in that sorrow all his own.

Still is that patient Head in love reclining
 When evening hangs her silver lamps on high;
And still, when morning in the East is shining,
 That great white wondrous Figure marks the sky.

*L. E. Kennaway.*

Place, O Lord, on my shoulders that most Divine Cross whose breadth is charity, whose length is eternity, whose height is omnipotence, and whose depth is unsearchable wisdom; fasten to it my hands and my feet, and conform Thy servant wholly to Thy Passion, O Lord.—*S. Bernard.*

From first to last Christ's life was one of obedience, and obedience involved suffering. Fix your mind for one moment on our Lord and on Him alone. Try and hear Him speaking to you individually from His Cross. Ask Him to reveal to you the message for your soul in each word He spoke, say to Him, "Speak, Lord, for Thy servant heareth." Christ died on Calvary "because He loved men, and because men did not love Him." "Greater love hath no man than this, that a man lay down his life for his friends;" but "God commendeth His love toward us, in that, while we were yet sinners, Christ died for us." Our Lord gave Himself to the misery and shame for love of us "while we were yet sinners." There, as he hangs upon the Cross with outstretched arms, He makes His great appeal to men: as Our Redeemer He cries out for the responsive love of men. Let us pray, then, to-day that the love of Christ in His Passion may draw us nearer to Himself; let us ask ourselves at the foot of the Cross, Have I really yet begun to love God, to love Jesus Christ? God does not ask us to go out of our way to court suffering. But He would have us accept, as loyal children, whatever He as a loving Father sends us.—*Randolph.*

If works of faith, and labours sweet of love,
 May not be mine, yet patient hope can be
Within my heart, like a bright censer's fire,
 With incense of thanksgiving mounting free.

Thou art our pattern to the end of time,
 Oh Crucified! and perfect is Thy will;
The workers follow Thee in doing good,
 The helpless think of Calvary, and are still.

*C. M. Noel.*

### Notes and Queries.

**FOR REGULATIONS SEE PAGE 136.**

*Visiting Nurses.*

(99) I should be grateful if you could give me the address of the hon treasurer or secretary of the Marylebone Visiting Nursing Association.—*A. C.*

The hon. secretary is the Countess Dowager of Desart, 2 Upper Berkeley Street, W., and all complaints or recommendations should be addressed to her. Full details as to charges, etc. will be supplied by Nurse Thomas, 10 Montagu Place, W.

*Sanitary Inspector.*

(100) Will you kindly tell me where I can qualify as sanitary inspector, and also if it would be difficult to do so?—*Sanitary.*

Apply to the Secretary, the Sanitary Institute, Parkes Museum, Margaret Street, W., for particulars.

*Maternity.*

(101) Will you kindly tell me where I could train for three months by giving my services? I am a monthly nurse, and working for doctors who think I ought to train.—*A. L.*

You cannot be trained anywhere free. For list of fees and training schools, see the "Nursing Profession: How and where to Train." Published by the Scientific Press.

*Nursing Co-operative Home.*

(102) Will you kindly tell me if there is a nurses' co-operative nursing home in Blackpool, and the address?—*Ethel.*

There is no nursing association managed by a committee in Blackpool, and we do not give the names of private institutions. The matron of the Victoria Hospital, Blackpool, has private nurses on her staff; perhaps, she would kindly give you information on the subject.

*Lady Roberts' Nurses.*

(103) Would you kindly tell me to whom to apply about Lady Roberts' Fund for Nursing Sisters and Officers' Hospitals? Is maternity training one of the necessary qualifications?—*J. B.*

All vacancies are filled privately.

*Medical Missionary.*

(104) I should deem it a great favour if you could tell me how I could get a post as medical missionary abroad. I am a hospital nurse, with medical, surgical, and ophthalmic training.—*Nurse Edith E.*

Apply to the Zenana Bible and Medical Mission, 2 Adelphi Terrace, W.C.; the Regions Beyond Missionary Union, 40 to 55 Bow Road, E.; the Palestine and Lebanon Nurses' Mission, 143 Clapham Road, S.W.; the Mildmay Institutions, Conference Hall, Mildmay Park, N.; or the Church Missionary Society, 16 Salisbury Square, E.C.

*California.*

(105) 1. Will you kindly tell me if the climate of California is suitable for a convalescent consumptive young man? 2. Can you inform me if there are openings there for trained nurses?—*F. L. S.*

1. California is generally reputed excellent for persons with delicate chests. 2. Yes, but it will take time to make your way unless you have introductions.

*Registered Midwife.*

(106) Will you kindly inform me what steps I must take to become a registered midwife? I have been recommended to do so by several medical gentlemen for whom I work.—*Nurse Lillie.*

Write for particulars to the Secretary, the Central Midwives' Board, Privy Council Office, Whitehall, S.W.

*District Nursing.*

(107) I have received one year's general, one year's fever, as well as some maternity training. Am I eligible for the Jubilee Institute for training as a district nurse?—*Martha.*

Write to the General Superintendent, Queen Victoria's Jubilee Institute for Nurses, St. Katherine's Precincts, Regent's Park, N.W.

**Important Nursing Textbooks.**

"The Nursing Profession: How and where to Train." 2s. net; 2s. 4d. post free.

"A Handbook for Nurses." (New Edition). 5s. net; 5s. 4d. post free.

"The Human Body." 5s. post free.

"Ophthalmic Nursing." (New Edition). 3s. 6d. net; 3s. 10d. post free.

"Gynæcological Nursing." 1s. post free.

"Art of Feeding the Invalid." (Popular Edition). 1s. 6d. post free.

"Practical Hints on District Nursing." 1s. post free.

## Figures and Tables

**Fig. 1. f1:**
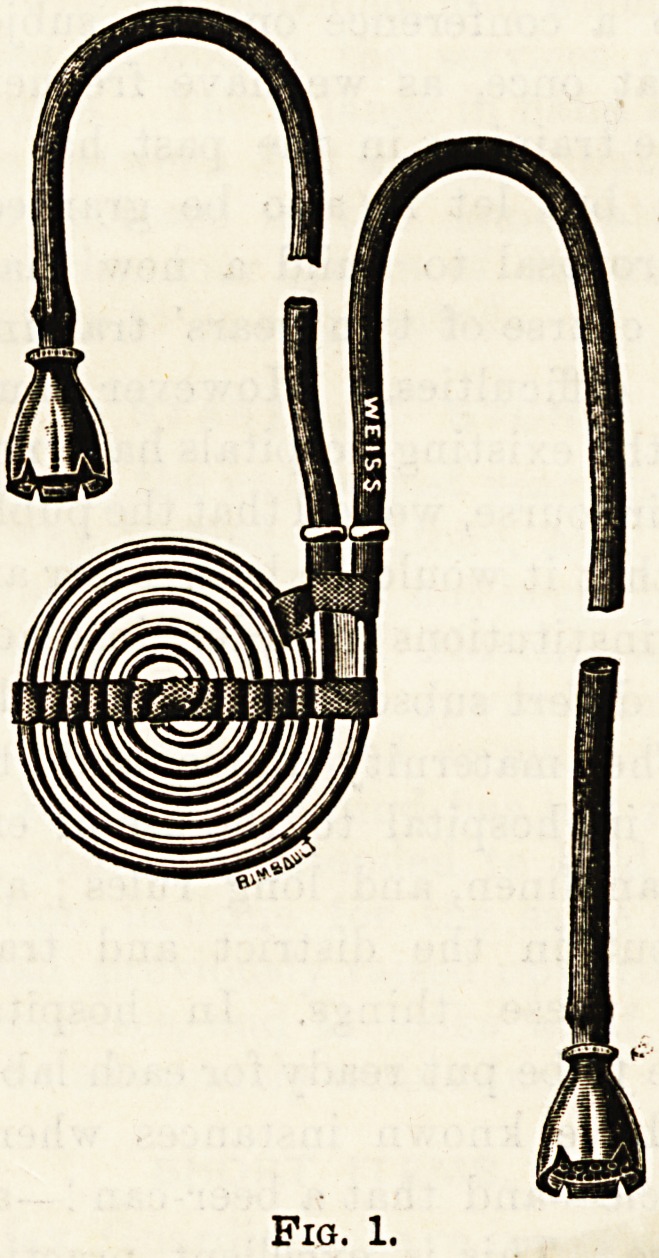


**Figure f2:**